# Operative strategies for ankylosing spondylitis-related thoracolumbar kyphosis: focus on the cervical stiffness, coronal imbalance and hip involvement

**DOI:** 10.1186/s12891-023-06810-2

**Published:** 2023-09-11

**Authors:** Wei Zhang, Wen Yin, Xilong Cui, Zihao Chai, Guohui Zheng, Ya Ding, Hongliang Wang, Yunlei Zhai, Haiyang Yu

**Affiliations:** 1 Department of Orthopaedics, Fuyang People’s Hospital Affiliated to Anhui Medical University, Anhui, 236000 China; 2 Clinical Research Center for Spinal Deformity of Anhui Province, Anhui, 236000 China

**Keywords:** Ankylosing spondylitis, Kyphosis, Surgical strategy, Classification

## Abstract

**Background:**

Cervical stiffness, coronal imbalance and limited hip movement all play crucial roles in designing the corrective surgery for ankylosing spondylitis-related thoracolumbar kyphosis (AS-TLK). However, a comprehensive classification and tailored strategies for directing clinical work are lacking. This study aims to investigate the types and surgical strategies for AS-TLK that consider cervical stiffness, coronal imbalance and hip involvement as the key factors.

**Methods:**

25 consecutive AS-TLK patients were divided into three types according to their accompanying features: Type I: with a flexible cervical spine; Type IIA: with a stiff cervical spine; Type IIB: with coronal imbalance; Type IIC: with limited hip movement. Type III is the mixed type with at least two conditions of Type II. Individual strategies were given correspondingly. Spinal-pelvic-femoral parameters were measured, Scoliosis Research Society outcome instrument-22 (SRS-22) was used and complications were recorded and analysed.

**Results:**

All patients (Type I 10, Type II 8 and Type III 7) underwent surgery successfully. 13 cases with 16 complications were recorded and cured. The patients were followed up for 24–65 months with an average of 33.0 ± 9.6 months. Both the sagittal and coronal parameters were corrected and decreased significantly (all, *p* < 0.05). SRS-22 scores showed a satisfactory outcome.

**Conclusion:**

Thoracolumbar kyphosis secondary to ankylosing spondylitis are complex and variable. Considering the factors of cervical stiffness, coronal imbalance and hip involvement assists in making decisions individually and achieving a desired surgical result.

## Introduction

Ankylosing spondylitis (AS), a chronic systemic immune and inflammatory-related disease may lead to motor dysfunctions in its advanced stage, predominantly characterized by spinal stiffness, kyphosis, scoliosis and limited hip movement [1–4]. AS-related thoracolumbar kyphosis would compromise patients’ appearance, vision field, cardiopulmonary and digestive functions. Osteotomy could be the only viable option to reconstruct spinal balance, enabling patients to stand and sit upright, gaze horizontally and lie flat [1, 2, 5–7]. Although performing osteotomies is no longer considered technically challenging [5, 7–9], the concomitant characteristics to be considered for designing preoperative planning remain unclear.

First, it is well known that a flexible cervical spine has a sufficient ability of compensation to maintain an ideal chin-brow vertical angle (CBVA). On the contrary, a stiff cervical spine requires a surgery rigorously, because inadequate or excessive osteotomy may lead to serious consequences, even a revision [10–13]. Second, a slight thoracolumbar scoliosis with a long arm of force but few compensatory curves often leads to an obvious deviation of central sacral vertical line (CSVL) [4]. Neglecting the coronal abnormality and solely correcting sagittal malalignment will make the coronal malformation prominent postoperatively [14, 15]. Third, 17–36% of AS patients exhibit varying degrees of hip joint involvement [16], which reduces the pelvis’s ability to adjust the relationship between the spine and lower limbs. Furthermore, the prone position required for surgery would become quite problematic for fused hip in a non-functional position with a pocketknife posture.

Several studies have demonstrated that cervical stiffness, coronal imbalance, and hip involvement directly impact surgical strategies and treatment outcomes [14, 17, 18]. However, most previous research [5–12, 17, 18] has focused on only one of the factors, with few exceptions. No comprehensive classification system exists to to unify the description of clinical characteristics and guide to make universal surgical strategies. Although Kim et al. [15] and Wang et al. [19] creatively proposed classifications based on the location of kyphotic apex, their focus on sagittal abnormalities alone limits their applicability to complex and variable AS-TLK. Our study, based on a series of cases from a single medical centre, aims to develop a novel classification system that focuses on cervical stiffness, coronal imbalance and hip involvement. To the best of our knowledge, this is the first comprehensive classification with individual surgical strategies given correspondingly.

## Materials and methods

### Patient demographics and radiographic parameters

Consecutive patients with AS-TLK who underwent spinal osteotomy surgery from March 2015 to August 2020 were reviewed retrospectively. The inclusion criteria were as follows: (1) AS diagnosis was made according to the New York Criteria [20]; (2) with thoracolumbar or lumbar kyphosis; (3) with definite indications and strong willingness for surgical correction; and (4) with a minimum of 2-year follow-up. Exclusion criteria included: (1) with only cervical or cervicothoracic kyphosis and (2) with definite contraindications or unwilling to undergo corrective surgery.

CBVA was measured by lateral photos with a natural standing position before and after operation. The cervical range of motion (ROM) referred to the difference of CBVA in hyperflexion and hyperextension position. Global kyphosis (GK), thoracolumbar kyphosis (TLK), lumbar lordosis (LL), sagittal vertical axis (SVA), femoral obliquity angle (FOA), T1 pelvic angle (TPA) and pelvic parameters (pelvic incidence, PI; pelvic tilt, PT) were measured by full-spine radiograph. Coronal Cobb angle (CCA) and CSVL were measured in cases with scoliosis present. Scoliosis Research Society outcomes instrument-22 (SRS-22) was used to evaluate the clinical outcome, and complications were recorded. Radiographic measurements were performed by Surgimap (Spine Software, version 2.0, New York, USA). The physical examination and radiographic measurements were evaluated by two senior physicians, and average values were taken.

### Types and surgical strategies

We classified the AS-TLK patients into three types according to the presence of cervical stiffness, coronal imbalance and hip involvement. Type II was further divided into three subtypes, each containing only one of the factors. Type I contained none of the three factors and type III contained two or more factors (Table 1).

**Table 1:** The descriptions of the types and surgical strategies

Type	Subtype	Total number	Cervical stiffness	Coronal imbalance	Hip involvement	Strategies
		25	8	6	10	
I	-	10	-	-	-	HP method
II	IIa	8	3	-	-	Modified CBVA method with or without HP method
	IIb		-	2	-	HP method and APSO
	IIc		-	-	3	HP method, Evaluating the state of hips, THA
III	-	7	5	4	7	All the above

*HP* Hilus pulmonis, *CBVA* Chin-brow vertical angle, *APSO* Asymmetric pedicle subtraction osteotomy, *THA* Total hip arthroplasty.

**Fig. 1:**
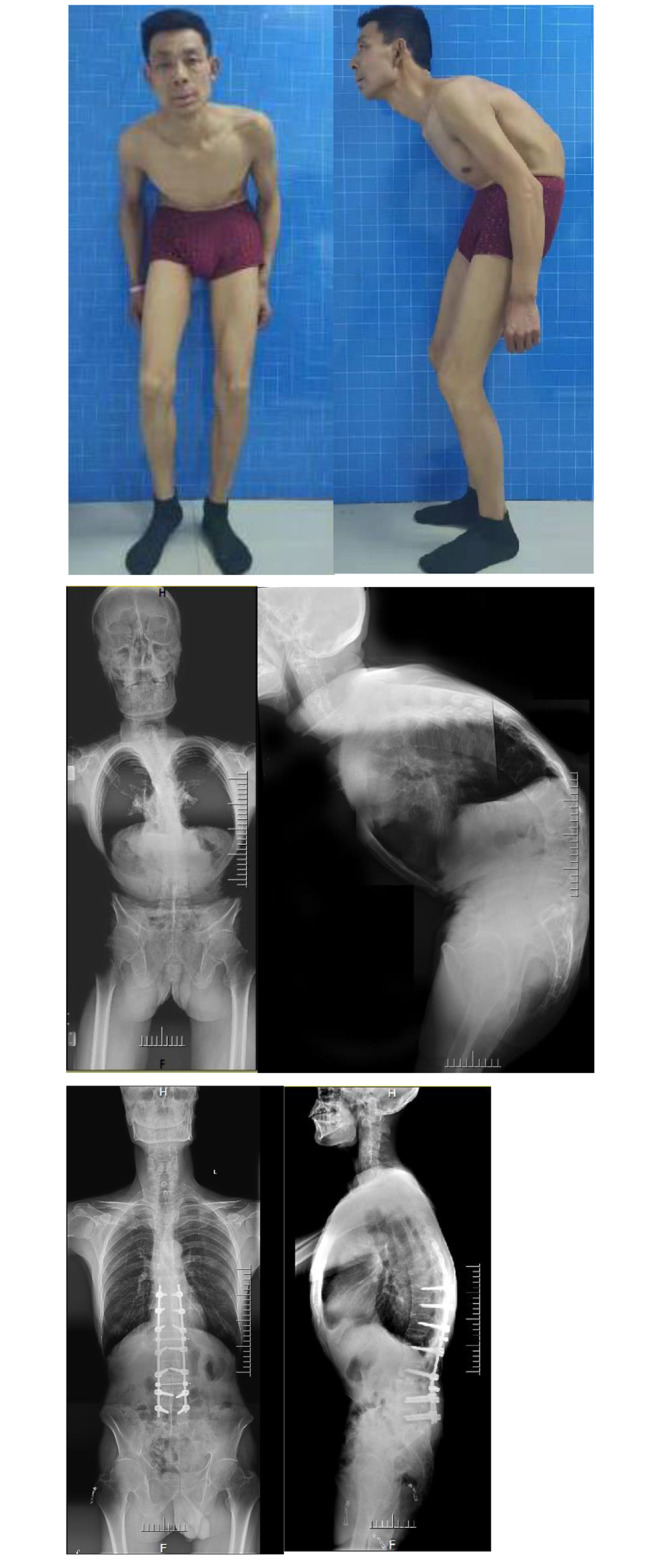
Type I: TLK-AS with a flexible cervical spine. The HP method was used without consdering the CBVA. Significant improvements with a satisfactory result could be seen comparing the photos and radiographs before operation and 36 months after operation

Type I: Cervical ROM ≥ 30°, with or without mild cervical or cervicothoracic kyphosis, without any condition of the three factors. Generally, type I patients could gaze horizontally preoperatively and have the best indication for the application of hilus pulmonis (HP) method [21] for preoperative planning. Shifting the trunk’s centre of gravity over the hip axis with osteotomy to complete sagittal reconstruction might be the only demand, with CBVA rarely needing consideration [21, 22] (Fig. 1).

Type IIA: Cervical ROM < 30°.

For patients without significant cervical or cervicothoracic kyphosis, the modified CBVA method, which considers optimal CBVA demand [23] and the offset of pelvic tilt [21, 24], was used to make preoperative planning.

**Fig. 2:**
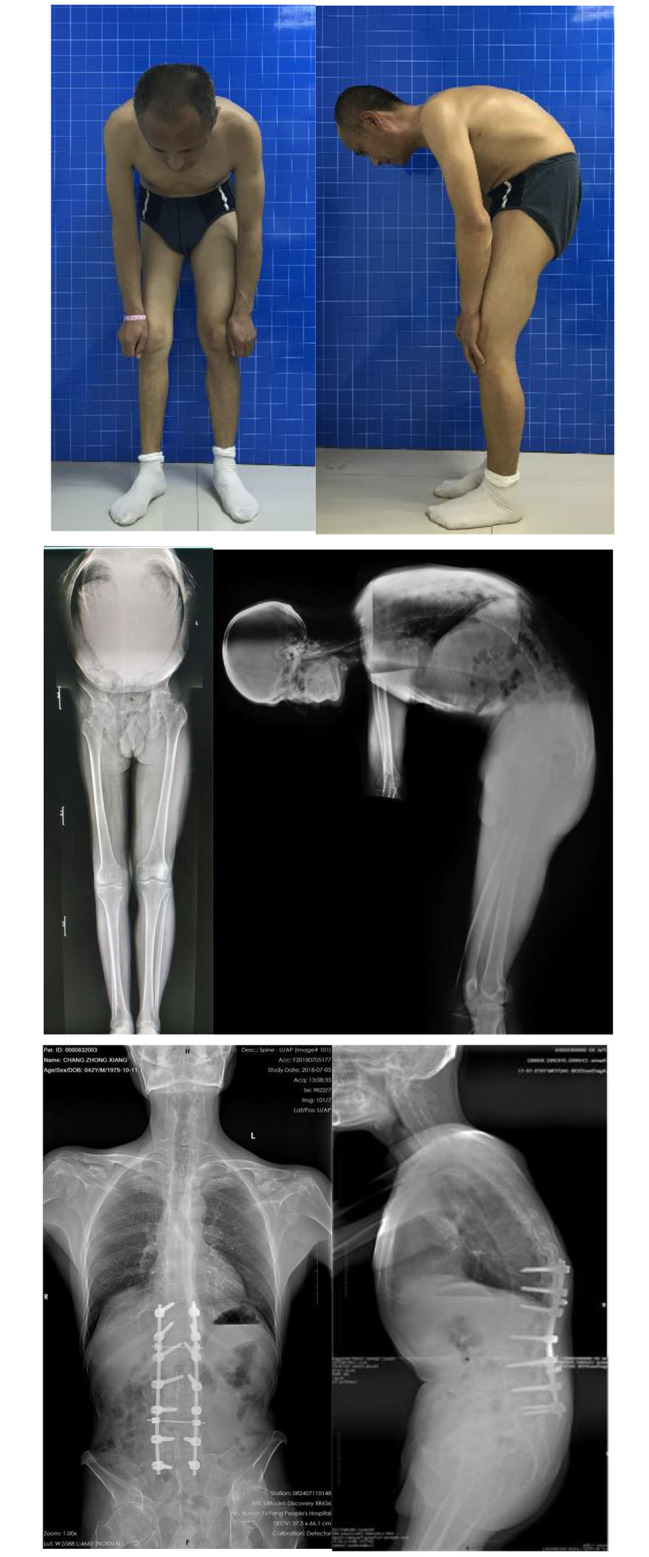
Type IIA: TLK-AS with a stiff cervical spine. The modified CBVA method was used. A good vision and the centre of gravity over the hip axis were achieved comparing the photos and radiographs before operation and 24 months after operation

For patients with significant cervical or cervicothoracic kyphosis, thoracolumbar kyphosis was corrected according to HP method first, followed by cervicothoracic osteotomy with the modified CBVA method (Fig. 2).

**Fig. 3:**
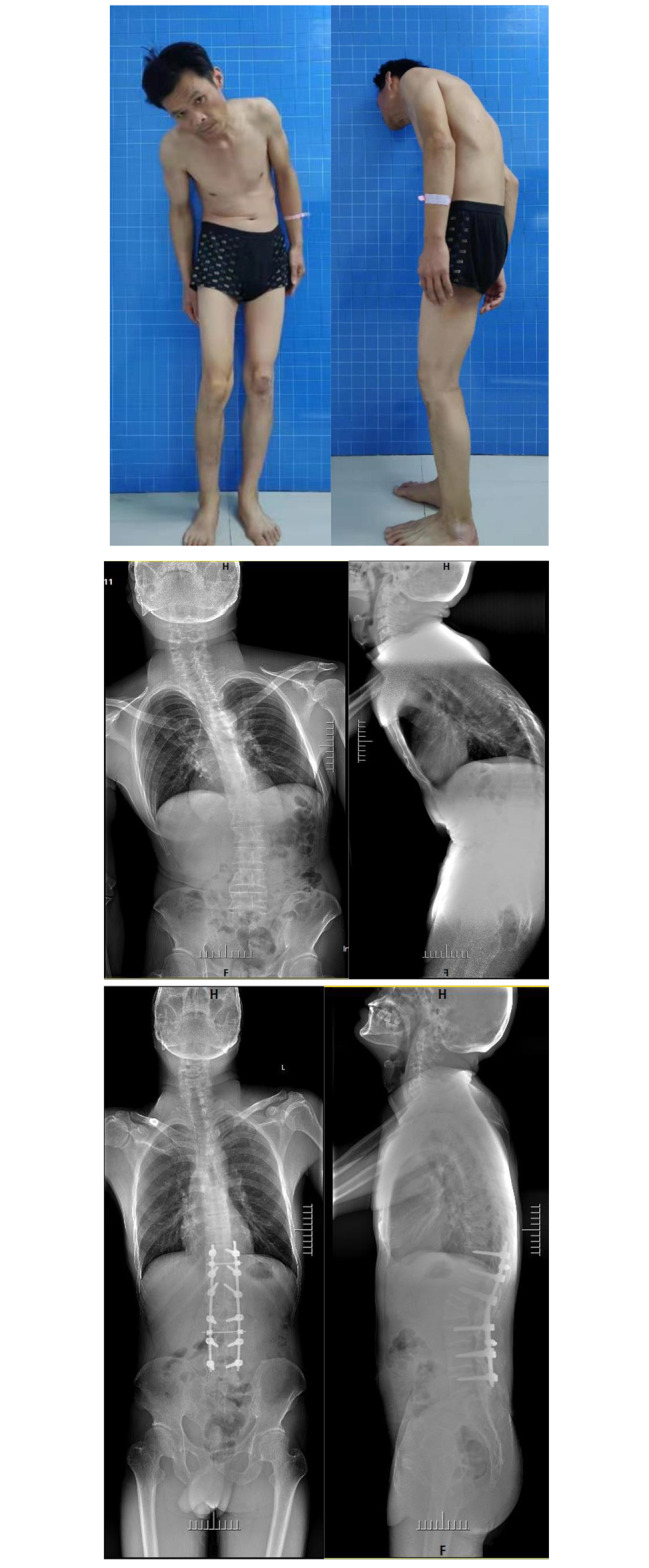
Type IIB: TLK-AS with an scoliosis of 20.3° and CSVL of 84 mm. The HP method combined with APSO was used. The photos and radiographs before operation and 36 months later postoperatively were compared and showed a good biplane reconstruction

Type IIB: With coronal imbalance. HP method [21] and combined sagittal–coronal correction with asymmetric osteotomy technique were applied to reconstruct sagittal and coronal balance [14] (Fig. 3).

Type IIC: With one or two hips flexing and extending ROM < 90°. Although HP method might still be effective, pathological position of hip joints and the effect after spinal correction surgery should be evaluated individually. For patients with hip hyperextension, the flexion activities would be relatively limited postoperatively. For patients with hip hyperflexion, total hip replacement (THA) may be required.

**Fig. 4:**
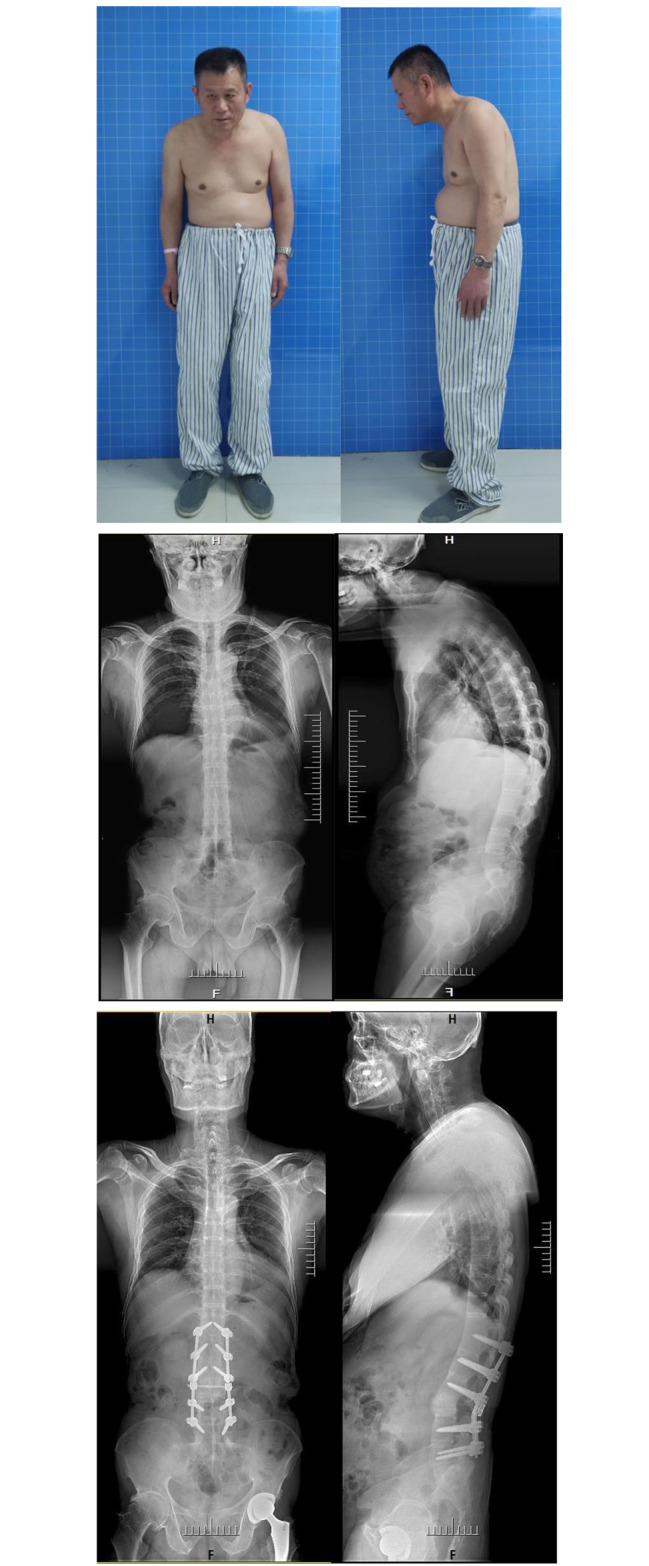
Type IIC: TLK-AS with the left hip involved seriously. Pathological state of the hip was well evaluated. The HP method combined with left THA was used. The photos and radiographs before operation and 39 months later postoperatively were compared and showed a good result

For patients with fused hip in non-functional flexion position, femoral neck amputation was conducted first, followed by spinal osteotomy in prone position and total hip arthroplasty [6] (Fig. 4).

**Fig. 5:**
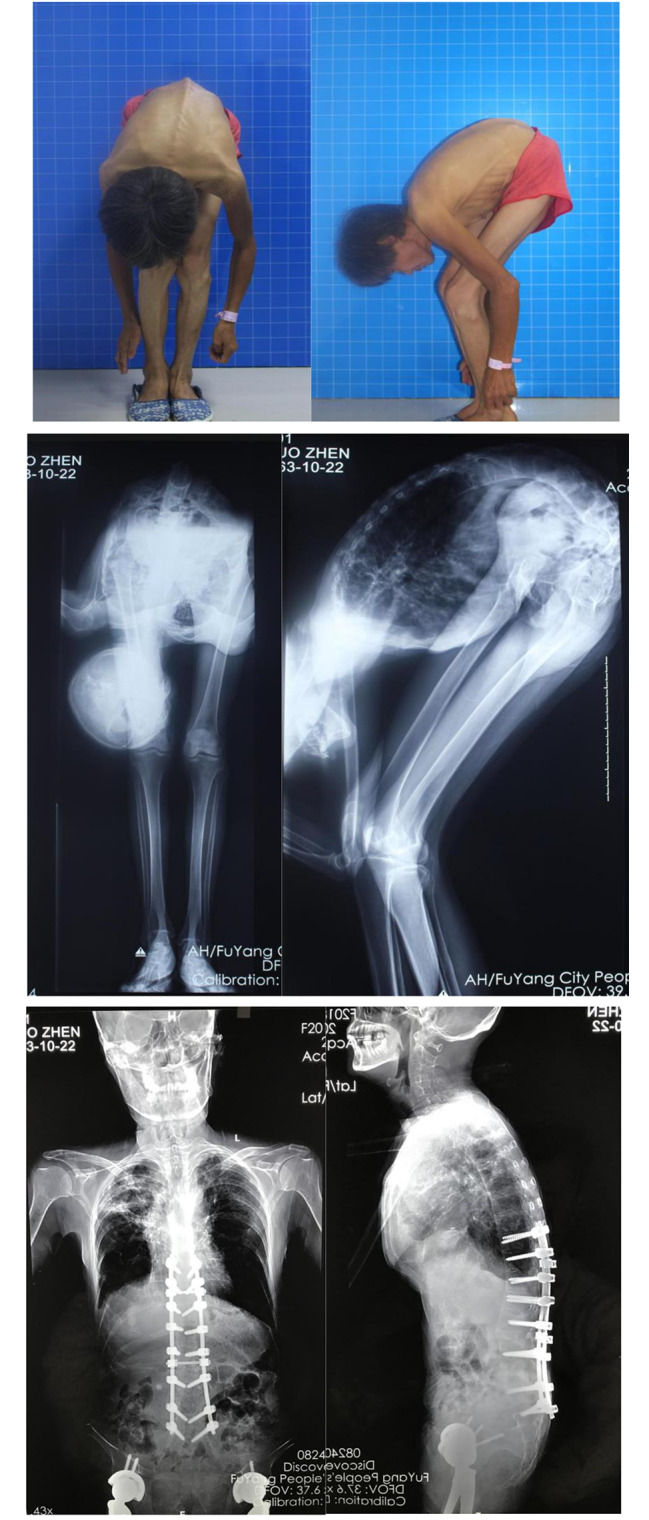
Type III: AS-TLK with cervical ROM of 10° and two fused hips in non-functional flexion position. Staged surgeries were carried out with a comprehensive method. The photos and radiographs before operation and 27 months later postoperatively were compared and showed a satisfactory result

Type III: With a mixed malformation involving at least two conditions of type II. As the most complex type of malformation, the key points and methods mentioned above should be considered and used comprehensively (Fig. 5).

### Summary of the surgical procedure

After the patient was placed on the adjustable prone positioning frame under general anaesthesia, a standard posterior midline approach was used to expose the posterior elements of the spine. Pedicle screws were inserted into two to four adjacent vertebrae cranial and caudal to the planned osteotomy site. The ventral sides of the lamina of the two ends were bitten to be thinner to decompress in advance. After placing a unilateral temporary rod, a single or two-level skipped pedicle subtraction osteotomy (PSO) or vertebral column decancellation osteotomy was performed. An assistant lowered the other modules in a targeted manner from the apical area to the sides [6]. Thus, the ends of the osteotomy came together and tended to close spontaneously. Residual clearance was totally closed with the force of screw and rod system. Posterolateral fusion was performed, and C-arm was used to confirm that the accomplishment of correction was in line with the plan. Somatosensory- and motor-evoked potentials were monitored throughout every procedure.

### Statistical analysis

Statistical analyses were performed with SPSS 22.0 software (IBM Corp.). Statistical significance was defined as *P* < 0.05. The data were analysed by Shapiro–Wilk test in accordance with normal distribution and expressed by mean and standard deviation. Spinal-pelvic-femoral parameters and SRS-22 scores of pre-operation and last follow-up were compared with the paired *t* test.

## Results

### Demographic and surgical characteristics

A total of 25 patients were included in the study (Table 1). Ten patients presented with cervical ROM ≥ 30° (type I, 40%), eight patients exhibited one of the three factors (type II, 32%), and seven patients displayed a mixed malformation involving at least two conditions of type II (type III, 28%). One case involved severe cervicothoracic kyphosis and three cases featured four fused hips in non-functional flexion position. All patients underwent spinal surgery in the prone position. A total of 45 osteotomies were performed, including one posterior osteotomy at C6/7, four single level osteotomy and 20 skipped two level osteotomies at thoracolumbar or lumbar region. The patients were followed up for 24–65 months, with an average of 33.0 ± 9.6 months. Fusions were obtained at the osteotomy site in all patients. Both the sagittal and coronal parameters were corrected and decreased significantly (all, *P* < 0.05, Table 2) when we compared the data obtained before operation with that obtained at last follow-up. SRS-22 scores showed a satisfactory outcome at the time of 2 years after surgery (all, *P* < 0.05, Table 3).

**Table 2:** Clinical parameters of pre-, postoperation and last follow-up

	Preoperation	postoperation	Last follow-up	P-Value*
CBVA(°)	56.4 ± 34.8	7.1 ± 6.6	7.8 ± 6.9	0.000
GK(°)	82.6 ± 24.0	18.5 ± 10.4	20.1 ± 10.6	0.000
TLK(°)	42.6 ± 14.9	13.2 ± 8.9	14.0 ± 8.7	0.000
LL(°)	15.9 ± 27.0	-32.6 ± 20.2	-32.1 ± 19.5	0.000
SVA(mm)	259.3 ± 97.0	94.8 ± 36.9	96.4 ± 37.0	0.000
CCA(°)	22.9 ± 8.4	6.3 ± 4.9	7.3 ± 5.4	0.003
CSVL(mm)	73.5 ± 25.3	27.7 ± 19.1	29.8 ± 19.7	0.008
FOA(°)	69.9 ± 27.5	31.9 ± 13.6	32.8 ± 13.5	0.000
TPA(°)	24.8 ± 8.3	7.5 ± 4.6	8.5 ± 4.8	0.000

*CBVA* Chin-brow vertical angle, *GK Global kyphosis*, *TLK* Thoracolumbar kyphosis; *LL* Lumbar lordosis, *SVA* Sagittal vertical axis, *CCA* Coronal Cobb angle, *CSVL* Central sacral vertical line, *FOA* Femoral obliquity angle, *TPA* T1 pelvic angle, *mm* millimeter. *: Compared peroperation with the last follow-up.

**Table 3:** SRS-22 scores at preoperation and 2 yeras after operation

	Preoperation	last follow-up	P-Value
Activity	1.5 ± 0.6	4.3 ± 0.6	0.000
Pain	1.7 ± 0.7	4.5 ± 0.5	0.000
Self imagine	1.4 ± 0.6	4.8 ± 0.4	0.000
Mental health	1.5 ± 0.6	4.4 ± 0.6	0.000
Satisfactory	-	4.5 ± 0.6	-

### Complications

There were 13 cases with 16 complications (Table 4), including tensional pain of abdomen, superior mesenteric artery syndrome, weak quadriceps, rod broken, radiculopathy and dural tear, which were cured conservatively after symptomatic treatments. The patient with chin-on-chest deformity suffered from gastric ulcer 3 days after posterior cervical osteotomy and received perforation repair surgery immediately with no serious consequences. One patient who was accidentally found rod broken without any symptoms during follow-up, also achieved bony fusion. Another slender patient developed an internal instrument-related skin ulcer 27 months after the spinal surgery and was successfully treated by partial removal of the instrumentation.

**Table 4:** Summary of surgery-related complications

Complications	NO.
Dural tear	4
Tensional pain of abdomen	6
Superior mesenteric artery syndrome	1
Weak quadriceps	1
Radiculopathy	1
Gastric ulcer	1
Rod broken	1
Instrument related skin ulcer	1

## Discussion

The natural history of AS shows that ossification and bridging occurred between vertebrae, followed by spinal stiffness, thoracolumbar kyphosis, loss of lumbar lordosis and disability to gaze horizontally. Then the trunk’s centre of gravity shifts anteriorly leading to muscle fatigue, low back pain and a stooped shape. Corrective surgery becomes inevitable when the temporary and vulnerable balance is broken [6–8, 10, 12, 17, 22, 23]. Numerous clinical characteristics, such as the location of kyphotic apex and radiological parameters, have been regarded as crucial for AS-TLK classification and surgery. The current study proposes the first comprehensive classification focused on cervical stiffness, coronal imbalance and hip involvement, with the following rationale.

### The role of cervical ROM

Almost all kinds of life in nature, including those with physical disabilities, engage in daily activities with a horizontal vision to the extent possible. Reconstructing sagittal vision horizontally is the primary task to correct AS-TLK deformity, and CBVA is the most important factor for preoperative planning. Suk et al. [12] first reported using the CBVA method to design an osteotomy strategy for patients with cervical stiffness and then Song et al. [23] found that CBVA with 10° to 20° was optimal for both appearance and inside activities. Furthermore, it is essential to remember that the pelvis rotates anteriorly around the femoral head to an almost zero position after AS-TLK correction. The contribution of osteotomy to improve CBVA will be partially offset along with the pelvic anteversion [21]. Therefore, when calculating the osteotomy angle using modified CBVA method, the change value of PT should be added preoperatively for AS-TLK of type IIA.

In another study, Song et al. [22] proposed to correct AS-TLK with HP method without considering the state of CBVA, yet achieving favorable clinical outcome. The current study also finds that HP method is sufficient for AS-TLK of type I due to the ample compensatory capacity of the flexible cervical spine. AS-TLK combined with cervical or cervicothoracic kyphosis represents a unique condition, and staged surgery may be the best choice [25]. Previous studies confirmed that PT was determined by lumbar lordosis when the lumbar osteotomy was completed [18, 24]. In other words, the osteotomy of cervicothoracic kyphosis will be primarily used to improve the CBVA, making minimal contributions to change PT. Thus, staged cervicothoracic osteotomy is designed with modified CBVA method based on the new CBVA after thoracolumbar kyphosis correction.

### The role of scoliosis

In some AS patients, unbalanced muscles, ligaments and facet joint usage causes lumbar spine with residual mobility to lose balance, resulting in scoliosis [4]. Scoliosis in AS is different from that of adolescent idiopathic scoliosis, typically presenting as a long scoliosis with few compensatory curves and occurring at or caudal to the thoracolumbar region. Slight lumbar scoliosis with a long lever arm can lead to significant coronal imbalance and strabismus. Asymmetric osteotomy [26, 27] is the optimal technique for the treatment of AS-TLK with coronal imbalance. In our clinical practice, two points were worthy of attention: (1) Simultaneous coronal and sagittal osteotomy often results in difference between the upper and lower planes at the osteotomy site, which was a crucial reason for sagittal translation and potential nerve injury. (2) The correction of AS scoliosis does not depend on the displacement from musculoskeletal and segments to be fixed in accordance with the bending rods. Instead, the proximal trunk must be translated to the convex side to close the osteotomy gap after the pelvis held. Then, in-situ fixations are performed with the stress-free insertion of the rods to prevent iatrogenic spinal fracture and internal fixation loosening.

### The role of hip involvement

The design of AS-TLK osteotomy is not merely the calculation with geometric theory. Compared to the trigonometric function method [28], the improvements of HP method, ASKyphoplan and FBI technique [14, 22, 23, 29, 30] were the introduction of the pelvic parameters to demonstrate the interdependent relationship between the spine and the lower limbs. The premise of using these methods is that the acetabulum could rotate freely around the femoral heads. However, 40% of the patients in this study had one or two hips flexing and extending ROM < 90°, or even hip ankylosis. Consequently, the hip joint may not achieve an ideal spinal-pelvic-femoral match as planned after spinal corrective surgery [3, 31].

Generally, a hip joint deviates from zero position when AS-TLK patients stand naturally. Limited mobility directly affects the acetabulum’s ability to rotate around the femoral head after spinal correction. Thus, the flexion and extension of a hip and its pathological position should be thoroughly evaluated for patients with hip involvement [31]. For patients with hip hyperextension, the pelvis rotating anteriorly after spinal correction invisibly occupies part of the initial effective flexion range, creating spaces for further hyperextension and then excessive osteotomy may lead to the phenomenon of ‘looking up’ due to the limited mobility of flexion. Conversely, for patients with hip hyperflexion, the potential capacity for further compensatory extension is theoretically large but usually lost. Postoperatively, the position of the hip joints will approach the zero position, reducing the range of further extension but increasing the range of flexion. As a result, insufficient osteotomy may lead to poor outcome due to the loss of compensatory extension [32]. In fact, patients with hip hyperflexion usually exhibit serious radiological changes of hip arthritis, even hip fusion. Spinal osteotomy alone cannot completely correct the deformity and total hip replacement may be required [25, 32]. For patients with fused hip in non-functional flexion position, we conducted femoral neck amputation first, followed by spinal osteotomy in prone position and total hip arthroplasty [6]. Another option is to perform spinal surgery first in the lateral position [13, 33].

### Limitations

Although we adopted all the above principles to develop the classification based on numerous previous studies for AS-TLK and the strategies for each type achieved good results in our clinical work, this study had several limitations. First, the number of clinical cases in our single centre was small, and this classification system had not been evaluated for its validity and specificity by other centres with larger samples. However, the classification based on our limited cases included almost all the common types of AS-TLK reported in the literatures. Second, in designing strategies for osteotomy, we mainly adopted HP and modified CBVA methods, which focused more on repositioning the centre of gravity over the hip axis and correcting the vision field, both paid less attention to the correction of SVA and TPA. As a result, the SVA of our cases reached 96 mm at the last follow-up, slightly higher than the 75 mm standard reported in the previous literature [34]. But it was also reported that the SVA within 100 mm had a good effect [15]. At the same time, the rigid thoracic kyphosis and limited hip movement reduced the improvement of TPA and FOA compared with that of other non-rigid spinal kyphosis [35, 36]. Additionally, the CBVA in our cases was ideal (Table 2). Third, we used a cervical ROM of 30° and hip ROM of 90° as the critical value mainly based on our limited experiences rather than a controlled study. Although the above ranges of mobilities were reported probably enough to meet the requirements of daily activities [3, 33], the exact ranges are worth further study. Fourth, we did not particularly address the treatment of thoracic kyphosis in the classification. As the fused thoracic and costal joints are very rigid, performing osteotomy at the middle thoracic region usually comes with a high risk and relatively poor ability to correct kyphosis. Cervicothoracic and proximal thoracolumbar osteotomies might be sufficient to correct the CBVA and SVA result from cervicothoracic and thoracic kyphosis [14, 37].

## Conclusion

AS-TLK are complex and variable. Considering the factors of cervical stiffness, coronal imbalance and hip involvement assists in making individual decisions and achieving the desired result for corrective surgery. The HP and modified CBVA method should be used under different conditions, taking into account whether the cervical ROM is ≥ 30° or not. Correcting coronal imbalance simultaneously with asymmetric osteotomy technique helps achieve better clinical outcomes. The postoperative influence should be thoroughly evaluated preoperatively when a hip ROM < 90°.

## Data Availability

The datasets supporting the findings of this study available from the corresponding author (YHY, fy.yhy@163.com) on reasonable request.

## Abbreviations

AS: Ankylosing spondylitis
AS-TLK: Ankylosing spondylitis-related thoracolumbar or lumbar kyphosis
CBVA: Chin-brow vertical angle
CSVL: Central sacral vertical line
ROM: Range of motion
GK: Global kyphosis
TLK: Thoracolumbar kyphosis
LL: Lumbar lordosis
SVA: Sagittal vertical axis
PI: Pelvic incidence
PT: Pelvic tilt
CCA: Coronal Cobb angle
SRS-22: Scoliosis Research Society outcomes instrument-22
HP: Hilus pulmonis
THA: Total hip replacement
FOA: Femoral obliquity angle
TPA: T1 pelvic angle
